# Death of Dr. Pereira

**Published:** 1853-04

**Authors:** 


*Death of Dr. Pereira.*— Died at London, Jan. 20th, Jonathan Pereira M. D., aged 49.

The name of Dr. Pereira is familiar to the profession of this country by his most valuable works on food and diet, and the materia medica. Science has lost an able and industrious laborer.

